# An investigation of WNT pathway activation and association with survival in central nervous system primitive neuroectodermal tumours (CNS PNET)

**DOI:** 10.1038/sj.bjc.6604979

**Published:** 2009-03-17

**Authors:** H A Rogers, S Miller, J Lowe, M-A Brundler, B Coyle, R G Grundy

**Affiliations:** 1 Children's Brain Tumour Research Centre, Queen's Medical Centre, University of Nottingham, D Floor Medical School (D32), Nottingham NG7 2UH, UK; 2 Department of Neuropathology, Nottingham University Hospital, Queen's Medical Centre, Nottingham NG7 2UH, UK; 3 Department of Pathology, Birmingham Children's Hospital, Birmingham B4 6NH, UK

**Keywords:** CNS PNET, sPNET, medulloblastoma, WNT pathway, *β*-catenin, cyclin D1

## Abstract

Central nervous system primitive neuroectodermal tumours (CNS PNET) are high-grade, predominantly paediatric, brain tumours. Previously they have been grouped with medulloblastomas owing to their histological similarities. The WNT/*β*-catenin pathway has been implicated in many tumour types, including medulloblastoma. On pathway activation *β*-catenin (CTNNB1) translocates to the nucleus, where it induces transcription of target genes. It is commonly upregulated in tumours by mutations in the key pathway components *APC* and *CTNNB1*. WNT/*β*-catenin pathway status was investigated by immunohistochemical analysis of CTNNB1 and the pathway target cyclin D1 (CCND1) in 49 CNS PNETs and 46 medulloblastomas. The mutational status of *APC* and *CTNNB1* (*β-catenin*) was investigated in 33 CNS PNETs and 22 medulloblastomas. CTNNB1 nuclear localisation was seen in 36% of CNS PNETs and 27% of medulloblastomas. A significant correlation was found between CTNNB1 nuclear localisation and CCND1 levels. Mutations in *CTNNB1* were identified in 4% of CNS PNETs and 20% of medulloblastomas. No mutations were identified in *APC*. A potential link between the level of nuclear staining and a better prognosis was identified in the CNS PNETs, suggesting that the extent of pathway activation is linked to outcome. The results suggest that the WNT/*β*-catenin pathway plays an important role in the pathogenesis of CNS PNETs. However, activation is not caused by mutations in *CTNNB1* or *APC* in the majority of CNS PNET cases.

The most common solid tumours during childhood are those of the central nervous system. Central nervous system primitive neuroectodermal tumours (CNS PNET) are high-grade embryonal tumours that occur at any extracerebellar site in the central nervous system and are composed of undifferentiated or poorly differentiated neuroepithelial cells (Louis *et al*, 2007). Currently, outcome for children with CNS PNET is poor with a relatively low overall 5-year survival rate (Reddy *et al*, 2000; Geyer *et al*, 2005; Timmermann *et al*, 2006; Johnston *et al*, 2008). Relatively little research has been undertaken to elucidate the molecular basis of CNS PNETs. Earlier they have often been grouped with the histologically similar tumour medulloblastoma; both being composed of poorly differentiated round ‘blue’ cells with scant cytoplasm (Louis *et al*, 2007). An increased understanding of CNS PNET biology will allow a more targeted approach to therapy.

Many studies have shown deregulation of developmental signalling pathways involved in normal brain development in medulloblastoma. Similar pathways are likely to be involved in CNS PNETs. The WNT/*β*-catenin signalling pathway plays a key role in many cellular functions related to tumourigenesis, including cell proliferation, differentiation and migration. It was originally linked to medulloblastoma through studies of Turcot syndrome, in which germline mutations in the *APC* gene have been identified (Hamilton *et al*, 1995).

*β*-catenin (CTNNB1) is the key downstream effecter of the pathway. When the pathway is inactive, CTNNB1 is bound in the cytoplasm to a complex containing the proteins adenomatous polyposis coli (APC), axin1 and glycogen synthase kinase-3*β* (GSK-3*β*). Glycogen synthase kinase-3*β* phosphorylates CTNNB1 at specific serine and threonine residues, allowing the protein to be targeted for degradation through the ubiquitin–proteosome system (Morin, 1999). On pathway activation the protein complex is destabilised, preventing phosphorylation and enabling CTNNB1 to translocate to the nucleus, where it acts as a co-activator of TCF and LEF transcription factors and leads to the upregulation of target genes, including *MYC* and *cyclin D1*(*CCND1*) (He *et al*, 1998; Tetsu and McCormick, 1999).

Activating mutations in *CTNNB1* have been identified in many different cancers, including colon, gastric, hepatocellular, prostate and Wilms' tumour (Morin *et al*, 1997; Iwao *et al*, 1998; Voeller *et al*, 1998; Koch *et al*, 1999; Koesters *et al*, 1999; Ogasawara *et al*, 2006). Single base substitutions have been identified at codons, in exon 3 of the gene, encoding serine and threonine residues targeted by GSK-3*β*, or at adjacent residues. These mutations are predicted to prevent phosphorylation and subsequent degradation of CTNNB1. Pathway activation through the stabilisation and nuclear accumulation of CTNNB1 has been shown in sporadic medulloblastomas (Eberhart *et al*, 2000; Koch *et al*, 2001; Yokota *et al*, 2002; Ellison *et al*, 2005; Clifford *et al*, 2006; Gajjar *et al*, 2006; Thompson *et al*, 2006). In the majority of cases this was caused by activating mutations in *CTNNB1*. A small study identified a single mutation in *CTNNB1* in one out of four CNS PNETs (Koch *et al*, 2001). No further research has been undertaken in CNS PNET to date.

*APC* is also commonly mutated in many tumour types including colon and gastric, with the majority of mutations occurring in the mutation cluster region (Miyoshi *et al*, 1992; Ogasawara *et al*, 2006). Mutations in *APC* are commonly truncating, resulting in proteins that are not able to form the cytoplasmic complex to target CTNNB1 for degradation. *APC* mutations are rare in sporadic medulloblastomas (Huang *et al*, 2000; Koch *et al*, 2001; Ellison *et al*, 2005; Clifford *et al*, 2006; Thompson *et al*, 2006). To date, only one study investigating *APC* mutational status in four CNS PNET tumours has been reported, in which no mutations were found (Koch *et al*, 2001).

A significant association between CTNNB1 nuclear immunoreactivity and survival has earlier been demonstrated in medulloblastoma, with nuclear accumulation being associated with a favourable outcome (Ellison *et al*, 2005; Gajjar *et al*, 2006). This is in contrast to other tumour types, such as colon, breast and hepatocellular carcinomas, in which nuclear immunoreactivity has been associated with disease progression and a poorer prognosis (Lin *et al*, 2000; Inagawa *et al*, 2002; Bondi *et al*, 2004).

The aim of this study was to investigate the WNT/*β*-catenin pathway in a set of CNS PNETs, using immunohistochemistry (IHC) to determine the cellular location of CTNNB1. This serves as a marker for pathway status, in which nuclear staining represents the active state and cytoplasmic the inactive state (Eberhart *et al*, 2000). The pathway target CCND1 was also investigated by IHC and results correlated with CTNNB1 localisation. MKI67 (antigen identified by monoclonal antibody to Ki67) protein levels were investigated to measure cell proliferation rates and compared with CTNNB1 and CCND1 data. The mutational status of exon 3 of *CTNNB1* and the mutation cluster region of *APC* were investigated by sequencing and correlated with the IHC results. The pathway status was also investigated in a set of medulloblastomas for comparison. Results were correlated with clinical information.

## Materials and methods

### Sample information

Tumour samples were obtained from the Children's Cancer and Leukaemia Group (CCLG) and the Cooperative Human Tissue Network (CHTN). A total of 25 snap-frozen CNS PNETs, all located in the cerebral hemispheres, and 22 medulloblastomas were obtained. Five CNS PNETs were recurrences, four with the paired primary. Two medulloblastomas were recurrences and one was paired. Eight pineoblastomas were also obtained, six were primary and two were recurrences (unpaired). Of the primary medulloblastomas, 85% were classical, 10% desmoplastic and 5% anaplastic. The recurrent tumours included one classical and one desmoplastic tumour. Medulloblastoma subtypes were assigned according to the WHO criteria (Louis *et al*, 2007). Two CNS PNETs and four pineoblastomas were obtained from CHTN. All other tumours were obtained from CCLG. When cutting a piece of frozen tissue for analysis, a small piece was taken and smeared along a slide, which was subsequently stained with haematoxylin and eosin to determine whether the tissue contained tumour cells.

Forty-two CNS PNETs (all cerebral), 46 medulloblastoma and seven pineoblastoma samples were fixed in 4% phosphate buffered formaldehyde and embedded in paraffin. Seven CNS PNETs were recurrences, five with the paired primary. Three medulloblastomas were recurrent tumours, one paired and two not paired. Of the primary medulloblastomas, 44% were classical, 33% desmoplastic, 14% anaplastic, 7% large cell and one medullomyoblastoma. The recurrent tumours included one classical, one anaplastic and one medullomyoblastoma. Blood samples were received for five CNS PNETs, three medulloblastomas and two pineoblastomas. All paraffin tumour samples were obtained from CCLG.

Pineoblastomas were included in the study because of their histopathological similarities to other CNS PNETs (Louis *et al*, 2007). In the UK, pineoblastomas are also treated with protocols similar to CNS PNETs (Pizer *et al*, 2006). For analysis they were included in the CNS PNET cohort.

Clinical information, including gender, age at diagnosis, time to recurrence, date of death or last follow-up if still alive and metastatic status (using the Chang staging system (Chang *et al*, 1969)), was obtained from CCLG and CHTN. Multiple Centre Research Ethics Committee approval was obtained for the study. Consent for use of tumour samples was taken in accordance with national tumour banking procedures and the human tissue act.

### Immunohistochemistry

Formalin fixed paraffin-embedded (FFPE) samples were analysed on a tissue microarray (TMA). After review by a pathologist, representative areas of tumour tissue were selected. Three cores from each tumour, taken from different locations in the section, were included on the array. Immunohistochemistry was carried out as described earlier (Ridley *et al*, 2008). Slides were incubated with either CTNNB1 (1 : 500, Cell Signalling Technology, Hitchin, UK), CCND1 (1 : 100, Abcam, Cambridge, UK) or MKI67 (MIB-1 clone, 1 : 50, Dako, Ely, UK).

Results for CTNNB1 were scored by location, either as nuclear (pathway active), cytoplasmic or negative (pathway inactive). Samples displaying nuclear staining were divided into two groups depending on the percentage of positive nuclei. Those with less than 10% of nuclei-positive were labelled as ‘low’ and those with greater than 10% as ‘high’. CCND1 and MKI67 were scored by calculating the percentage of positive cells. A total of 100 cells were counted in five randomly chosen fields of view. CCND1 was considered to be overexpressed if greater than 10% of cells were positive. Lost cores or those in which the majority of tissue was necrotic were removed from the analysis.

### Mutational analysis

DNA was extracted from 25 snap-frozen CNS PNETs, 22 medulloblastomas and eight pineoblastomas. The pineoblastoma samples were included in the CNS PNET cohort for analysis. Constitutional DNA from five blood samples from CNS PNET patients, three from medulloblastoma and two from pineoblastoma patients was also extracted. A total of 5–10 mg of tissue was lysed in lysis buffer (50 mM Tris pH 8, 100 mM EDTA pH 8, 100 mM NaCl and 1% SDS) and proteinase K (20 mg ml^−1^) at 37°C overnight. DNA was obtained by phenol:chloroform extraction followed by isopropanol precipitation. Standard PCR reactions were carried out using earlier published primers designed to amplify exon 3 of *CTNNB1* (Genbank accession number X89579) (Koch *et al*, 1999). A combination of published (Huang *et al*, 2000) and newly designed primers (5′ primer sequence TGCCACTTGCAAAGTTTCTTC, 3′ primer sequence CATTCCACTGCATGGTTCAC, annealing temperature 60°C) were used to amplify the mutation cluster region of *APC* (Genbank accession number NM000038). PCR products were purified by incubation with 0.3 U shrimp alkaline phosphatase (Promega, Southampton, UK) and 1.5 U exonuclease I (NEB, Hitchin, UK) at 37°C for 8 min followed by 15 min at 72°C. Sequencing reactions were performed on 1 *μ*l purified PCR product using Big Dye V1.1 (Applied Biosystems, Warrington, UK), following the manufacturer's protocol.

### Statistical analysis

Association between clinical factors and immunohistochemical status was investigated using the Fisher's Exact Test. Overall and progression-free survival were investigated using the Kaplan–Meier method. The differences were estimated using the log-rank (Mantel-Cox) test. Overall survival was defined as the time between date of original diagnosis and date of death. Progression-free survival was defined as the time between date of original diagnosis and date of first event (recurrence or death). Patients still alive at the end of the study were censored at the date of last follow-up. Median survival was estimated using Kaplan–Meier method.

## Results

### Clinical characteristics

**Figure 1:**
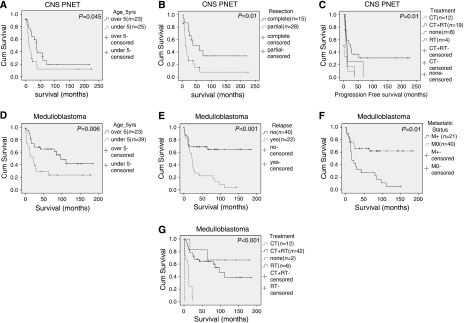
Kaplan–Meier curves for analysis of the CNS PNET and medulloblastoma patient cohorts. A significant difference in overall survival was seen between CNS PNET patients under the age of 5 years at diagnosis and those over 5 years (*P*=0.045) (**A**). Patients who had a complete rather than partial resection had a better prognosis (overall survival, *P*=0.01) (**B**). CNS PNET patients treated with both chemotherapy (CT) and radiotherapy (RT) had a significantly better progression-free survival (*P*=0.01) (**C**). Medulloblastoma patients under 5 years at diagnosis also showed a significant association with survival (overall survival, *P*=0.006) (**D**). Medulloblastoma patients that had not relapsed (*P*<0.001) (**E**) or metastasised (overall survival, *P*=0.01) (**F**) had a significantly better prognosis. Medulloblastoma patients treated with either radiotherapy or chemotherapy and radiotherapy also had a better outcome (overall survival, *P*<0.001) (**G**). All survival times are in months.

In total, the frozen and FFPE samples represented 43 patients with CNS PNET, 12 with pineoblastoma and 62 with medulloblastoma. The CNS PNETs and pineoblastomas were analysed as one cohort (CNS PNET cohort). The clinical characteristics of the two cohorts are summarised in Table 1. Age at diagnosis, sex, relapse, metastatic status, resection and treatment status were analysed for an association with survival in both cohorts. In the CNS PNET, cohort patients under 5 years had a significantly worse prognosis than the rest of the cohort (overall survival, *P*=0.045) (Figure 1A). Patients who had a complete rather than partial resection had a significantly better outcome (overall survival, *P*=0.01) (Figure 1B). Patients who were treated with both chemotherapy and radiotherapy had a better overall survival and significantly better progression-free survival (*P*=0.01) (Figure 1C). No other factors were significant in the CNS PNET cohort. In the medulloblastoma cohort age at diagnosis, relapse, metastatic status and treatment were significantly associated with survival. Patients under 5 years had a worse prognosis (overall survival, *P*=0.006) (Figure 1D). Patients who had relapsed (overall survival, *P*<0.001) or metastasised (overall survival, *P*=0.01) also had a poorer outcome (Figure 1E and F). Patients who had either radiotherapy or chemotherapy and radiotherapy had a better outcome (overall survival, *P*<0.001) (Figure 1G).

### CTNNB1 immunohistochemistry

**Figure 2:**
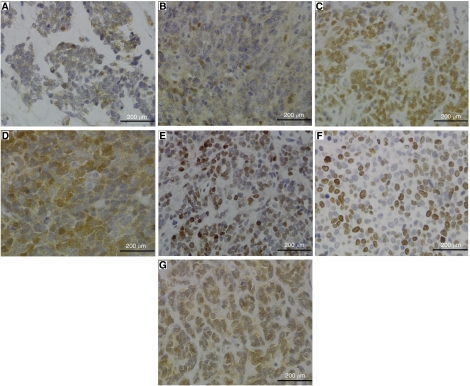
Immunohistochemical analysis of CTNNB1, CCND1 and MKI67 in the CNS PNET and medulloblastoma cohorts. Two patterns of CTNNB1 nuclear staining were seen in both CNS PNETs and medulloblastomas. A low level of nuclear staining (less than 10%) was seen in some CNS PNETs (**A**) and medulloblastomas (**B**). In others a high level of nuclear staining (greater than 10%) was seen (**C** CNS PNET and **D** medulloblastoma). Over expression of CCND1 was also seen in a subset of tumours (**E**, CNS PNET). MKI67 levels were measured in both cohorts (**F**, CNS PNET). Levels did not correlate with CCND1. An additional CNS PNET sample containing a mutation in CTNNB1 exon 3 was analysed in a separate experiment and displayed high CTNNB1 nuclear staining (**G**).

The cellular location of CTNNB1 was investigated in 49 tumours in the CNS PNET cohort (including 7 pineal tumours), which included 42 primary samples. Of 28 scorable primary CNS PNETs 10 displayed CTNNB1 nuclear staining (36%), which included one pineal tumour (out of five) (Table 2). Two patterns of nuclear staining were noted. In the first, only a small number of nuclei were positive for CTNNB1. In the second, a large number of nuclei were positive for CTNNB1 across the majority of the tissue analysed. For scoring, the groups were defined by the percentage of CTNNB1-positive nuclei, with the low nuclear group containing less than 10% of positive nuclei and the high nuclear group containing greater than 10% of positive nuclei. Six samples displayed high CTNNB1 nuclear staining, with four having greater than 30% of positive nuclei. In the other four (including the pineoblastoma), low CTNNB1 nuclear positivity was seen (Figure 2A and C). Cytoplasmic staining was seen in most tumours with only one tumour negative. Seven recurrences were also analysed, with six producing scorable results. Three displayed high CTNNB1 nuclear staining and three cytoplasmic staining. For two of the recurrences, the primary sample from the same patient was analysed. Both displayed the same staining patterns. Concordance of results across all cores was seen for all samples except one, in which low CTNNB1 nuclear staining was seen in only one core. The sample was scored as low nuclear CTNNB1.

CTNNB1 cellular location was also investigated in 46 medulloblastomas, including 43 primary samples. A total of 10 out of 37 scorable primary medulloblastomas displayed CTNNB1 nuclear staining (27%) (Table 2). The same pattern of high and low nuclear staining as the CNS PNET cohort was observed. High CTNNB1 nuclear staining was seen for three tumours and focal high CTNNB1 nuclear staining was seen for two tumours. All high CTNNB1 nuclear tumours displayed less than 30% of nuclei positive. Five tumours displayed low CTNNB1 nuclear positivity (Figure 2B and D). Four tumours were negative, the rest displayed cytoplasmic staining. A result for only one recurrent sample was obtained, which was negative for CTNNB1. The primary sample was not analysed. Concordance across all cores was seen for all medulloblastomas except four. Two displayed low CTNNB1 nuclear staining in two cores and only cytoplasmic staining in the other and were placed in the low CTNNB1 nuclear group. One sample displayed focal high CTNNB1 nuclear staining in two out of the three cores. In the other sample, focal high CTNNB1 staining was seen in one out of the three cores. The latter two samples were placed in the high CTNNB1 nuclear group.

A relatively high number of samples were unscorable, owing to core loss or the presence of necrotic tissue, in both CNS PNET and medulloblastoma cohorts. In the CNS PNET cohort, 28 primary and six recurrent tumours were scorable and 14 primary plus one recurrent tumour were unscorable. In the medulloblastoma cohort, 37 primary and one recurrent tumour were scorable and six primary plus two recurrent tumours were unscorable. Clinical features of scorable and unscorable tumours were examined to ensure there was no sampling bias (Supplementary Tables 1 and 2).

### CCND1 immunohistochemistry

CCND1 results were obtained for 27 primary tumours in the CNS PNET cohort. Where positive, the protein was localised in the nucleus. CCND1 was overexpressed in 12 samples (44%, no pineoblastomas) with percentages of positive cells ranging from 11 to 56% (Figure 2E). Of the 10 tumours with nuclear CTNNB1, eight (80%) displayed CCND1 overexpression, which included all tumours with high CTNNB1 nuclear positivity. A total of 4 out of 17 (24%) tumour samples displaying cytoplasmic or negative CTNNB1 staining also overexpressed CCND1. Six recurrent tumours were analysed, three with high CTNNB1 nuclear staining and three with CTNNB1 cytoplasmic staining. All six displayed CCND1 overexpression (Table 2). A significant correlation was found between CTNNB1 nuclear positivity and CCND1 overexpression in the primary CNS PNETs (Fisher's Exact Test, *P*=0.007).

In medulloblastoma, CCND1 overexpression (nuclear) was seen in 4 out of 37 (11%) primary tumours with percentages of positive cells ranging from 14 to 22%. This included 3 out of 10 (30%) tumours with CTNNB1 nuclear staining, one with low and two with a high level of staining. CCND1 was also overexpressed in 1 out of 27 (4%) tumours with cytoplasmic or negative CTNNB1 staining (Table 2). An association between CTNNB1 nuclear staining and CCND1 overexpression in the primary medulloblastomas just below significance was identified (Fisher's Exact Test, *P*=0.052). However, 70% of tumours with nuclear localisation of CTNNB1 did not display CCND1 overexpression.

As for CTNNB1 IHC, a relatively high number of samples were unscorable in the CCND1 analysis. For the CNS PNET cohort, one additional sample was unscorable in the CCND1 analysis compared with the CTNNB1 analysis. For medulloblastoma, the unscorable samples were the same in both experiments. Therefore, no new comparisons were made.

### MKI67 immunohistochemistry

MKI67, a proliferation marker, was investigated to see if CCND1 overexpression was affecting cell proliferation. MKI67 results were obtained for 18 primary and six recurrent tumours in the CNS PNET cohort. The number of positive cells ranged from 0 to 42% (Figure 2F). No correlation was found between MKI67 results and CCND1, or CTNNB1 localisation.

MKI67 results were obtained for 34 primary medulloblastomas and one recurrence. The number of positive cells ranged from 0 to 40%. No correlation was found between MKI67 results and CCND1, or CTNNB1 localisation.

The clinical features of the scorable and unscorable samples in the CNS PNET cohort were compared to check for sampling bias, with none found (Supplementary Table 3). The scorable and unscorable samples in the medulloblastoma cohort were very similar to the CTNNB1 and CCND1 analyses; therefore, no new comparison was made.

### Sequencing

**Figure 3:**
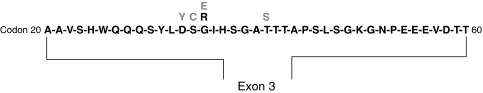
Schematic representation of mutation locations in exon 3 of *CTNNB1*. Amino acid substitutions are indicated above the sequence; grey changes represent mutations from medulloblastoma and black from CNS PNET.

In the CNS PNET cohort, only one of the 26 primary tumours sequenced contained a mutation in exon 3 of *CTNNB1* (4%) (Figure 3). No mutations were found in six recurrent samples. The mutation was a missense point mutation at codon 34 (GGA>CGA), converting glycine to arginine. No blood samples contained mutations. The matching blood sample for the tumour containing a mutation was not available for sequencing. An IHC result for the CNS PNET sample, for which a mutation in *CTNNB1* was found, was not obtained from the TMA due to core drop out. However, high CTNNB1 nuclear staining was seen in a separate experiment (Figure 2G). Four other primary and one recurrent tumour that displayed CTNNB1 nuclear staining were sequenced and none contained mutations (Table 2).

Four out of 20 primary medulloblastomas contained *CTNNB1* mutations (20%) (Figure 3). Four recurrent samples were sequenced with none containing mutations. All mutations were missense point mutations; one at codon 32 (GAC>TAC), converting aspartic acid to tyrosine; two at codon 33 (TCT>TGT), converting serine to cystine; and one at codon 34 (GGA>GAA), converting glycine to glutamic acid. One sample with a mutation at codon 33 also contained a missense point mutation at codon 40 (ACT>AGT), converting threonine to serine. No blood samples contained mutations. No blood samples from patients with mutations in their tumours were sequenced. There was only a small overlap in the cohorts of medulloblastoma samples analysed by IHC and sequencing. Therefore, none of the samples displaying CTNNB1 nuclear staining were sequenced and no IHC result was obtained for any of the tumours containing mutations (Table 2).

No mutations were found in the mutation cluster region of *APC* in 20 CNS PNET and 19 medulloblastoma primary tumours sequenced. None of the blood samples, from both tumour types, contained *APC* mutations.

### Clinical correlates

In the CNS PNET cohort, CTNNB1 nuclear cases contained a higher proportion of males (male: female ratio 4 : 1 compared with 0.6 : 1 in non-nuclear), and displayed a higher 5-year survival rate (30% compared with 13%) than the non-nuclear cases. However, no significant association was seen for any clinical factor tested (Fisher's Exact Test). Analysis could be limited by the small sample size (*n*=28).

**Figure 4:**
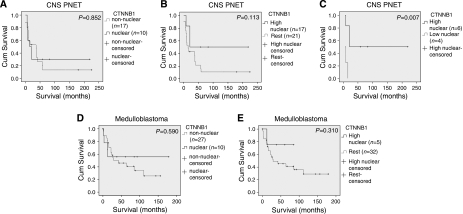
Kaplan–Meier curves for analysis of CTNNB1 IHC. Comparison of CNS PNETs displaying nuclear CTNNB1 with non-nuclear staining did not reveal a significant difference in overall survival (*P*=0.852) (**A**). Comparison of high CTNNB1 nuclear tumours (high nuclear) with the rest of the cohort (rest; tumours displaying low nuclear, cytoplasmic or negative staining), although not statistically significant (overall survival, *P*=0.113), suggested a trend towards a better prognosis for the high CTNNB1 nuclear group (**B**). A significant difference in overall survival was seen between CNS PNETs displaying high and low CTNNB1 nuclear staining (overall survival, *P*=0.007) (**C**). In the medulloblastoma cohort comparison of nuclear with non-nuclear CTNNB1 tumours was not significant (overall survival, *P*=0.590), but suggested a trend towards better survival for the nuclear group (**D**). Comparison of CTNNB1 high nuclear cases (high nuclear) with all other tumours in the medulloblastoma cohort (rest) also suggested the same trend (overall survival, *P*=0.310) (**E**). All survival times are in months.

Comparison of all CNS PNET CTNNB1 nuclear cases with non-nuclear cases did not reveal a significant difference in overall or progression-free survival (*P*=0.852 and 0.536, respectively) (Figure 4A). However, comparison of high CTNNB1 nuclear cases to all other tumours (low CTNNB1 nuclear plus cytoplasmic and negative cases), although not significant (overall survival, *P*=0.113), suggested a trend towards the association of high CTNNB1 nuclear staining with a more favourable outcome (Figure 4B). Comparison of cases with high CTNNB1 nuclear staining to just those with a low level of nuclear staining did reveal a significant difference in overall survival (*P*=0.007) (Figure 4C). However, only limited conclusions can be drawn because of the small number of samples analysed (*n*=10). The results were supported by the 5-year overall survival rates. Patients with a high level of CTNNB1 nuclear staining had a 5-year overall survival rate of 50% compared with 11% for the rest of the cohort.

In the medulloblastoma cohort, association between CTNNB1 nuclear immunoreactivity and percentage of cases that had relapsed almost reached significance (Fisher's exact test, *P*=0.056) with a lower percentage of relapses seen in the nuclear cases. Although not significant, there was a male bias in the CTNNB1 nuclear cases (male: female ratio 9 : 1 compared with 2.9 : 1). A total of 60% of CTNNB1 nuclear cases were desmoplastic compared with 31% of non-nuclear tumours. CTNNB1 nuclear immunoreactivity was not significantly linked to overall or progression-free survival (*P*=0.590 and 0.517, respectively). However, the Kaplan–Meier curves suggest a difference (Figure 4D). This was also reflected in the overall survival rates. At 5 years, 56% of patients with CTNNB1 nuclear staining and 46% of patients with only cytoplasmic or negative staining were still alive. At 10 years, the difference between survival rates was greater with 56% of CTNNB1 nuclear patients still alive and 24% of those with only cytoplasmic or negative staining. It is possible that significance was not reached because of the relatively small sample size in this study (*n*=37). Comparison of cases displaying a high level of CTNNB1 nuclear positivity to the rest of the cohort was not significant (overall survival, *P*=0.310), but suggested a better survival for the high nuclear group (Figure 4E). This was supported by the difference in 5-year overall survival rates of 80% for patients with high CTNNB1 nuclear staining compared with 44% for the rest of the cohort.

## Discussion

This study is the first to extensively investigate the status of the WNT/*β*-catenin pathway in CNS PNETs and has shown pathway activation in a high proportion of tumours (36%), as well as suggested a link between pathway activation and a more favourable outcome. The high percentage of tumours displaying activation suggests that the pathway plays an important role in the pathogenesis of CNS PNETs and is a potential target for future therapies. Further investigation is needed to validate findings and understand the biological role the pathway is playing in tumourigenesis. An equivalent rate of pathway activation was seen in the medulloblastomas investigated in this study (27%), in agreement with earlier research (Eberhart *et al*, 2000; Yokota *et al*, 2002; Ellison *et al*, 2005). Although a different CTNNB1 antibody was used in these studies (BD Transduction Laboratories, San Jose, CA, USA) the agreement in the results suggests the two alternative antibodies are comparable.

Nuclear localisation of CTNNB1 was used to determine pathway activation. The results were supported by the significant correlation with CCND1 overexpression in both cohorts. CCND1 has earlier been shown to be a target of the WNT/*β*-catenin pathway (Tetsu and McCormick, 1999). The evidence, although significant, was not as strong in the medulloblastoma cohort, with 70% of tumours displaying nuclear localisation of CTNNB1 showing no CCND1 overexpression. This included three out of five tumours with high nuclear CTNNB1 expression. Correlation of CCND1 overexpression and CTNNB1 nuclear localisation was not absolute in either cohort, with some tumours displaying only cytoplasmic or negative CTNNB1 staining overexpressing CCND1. This could suggest that an alternative factor is influencing CCND1 overexpression. CCND1 expression has been increased in other tumour types by gene amplification or translocation, or controlled by alternative cell signalling pathways such as the sonic hedgehog pathway (Fu *et al*, 2004; Marino, 2005). However, the significant correlation with CTNNB1 nuclear localisation found particularly in the CNS PNET cohort, strongly suggests that the WNT/*β*-catenin pathway is increasing CCND1 expression in the tumours with pathway activation in this study.

The correlation between CTNNB1 nuclear localisation and CCND1 overexpression suggests that WNT/*β*-catenin pathway activation is affecting cell proliferation. However, no correlation was found between CCND1 and MKI67 in either cohort. It may be that WNT/*β*-catenin pathway activation is not affecting cell proliferation or that alternative mechanisms are affecting the proliferation rate in tumours without pathway activation therefore masking any correlation that could be found.

Unlike medulloblastomas, pathway activation in CNS PNETs does not seem to be caused by mutations in exon 3 of *CTNNB1*, with only one CNS PNET in this study containing a mutation. This tumour did display high nuclear staining of CTNNB1, suggesting that the mutation could be the cause of pathway activation in this sample. The overlap in the cohorts used for IHC and sequencing was relatively low. Results for both methods were only obtained for 12 primary and two recurrent samples. This included four primary and one recurrent tumour with CTNNB1 nuclear staining. Six additional primary and two recurrent tumours with CTNNB1 nuclear staining were not sequenced. Therefore, no definite conclusions can be drawn about whether there is a correlation between *CTNNB1* mutation and nuclear staining. However, only one mutation was found in 32 tumours sequenced, which included 17 tumours with no IHC result, and therefore no known WNT/*β*-catenin pathway status, suggesting *CTNNB1* exon 3 mutation to be rare.

Only four medulloblastomas had both IHC and sequencing results, all displaying cytoplasmic CTNNB1 staining and containing no mutations in *CTNNB1* exon 3. Therefore, it cannot be concluded whether there is a correlation between nuclear staining and mutation of *CTNNB1*. However, the overall mutation rate identified (20%) was similar to those found earlier in which a correlation was reported, suggesting that this is likely to be the case in this study (Ellison *et al*, 2005; Clifford *et al*, 2006; Thompson *et al*, 2006).

Matching blood samples were not available for any of the CNS PNET or medulloblastoma tumours containing mutations therefore it is not known whether these are somatic or constitutional.

The mutations detected in the medulloblastomas are consistent with those described in earlier studies (Eberhart *et al*, 2000; Koch *et al*, 2001; Yokota *et al*, 2002; Ellison *et al*, 2005; Clifford *et al*, 2006; Thompson *et al*, 2006). The mutation in the single CNS PNET identified here is in the same codon as one of the medulloblastomas and a CNS PNET in an earlier study, in which a missense point mutation caused a glycine to valine substitution (Koch *et al*, 2001). The substitution of glycine to arginine, found in the CNS PNET in this study, has not been seen in CNS PNETs before, but has been found in medulloblastoma and pancreatic tumours (Abraham *et al*, 2002; Haberler *et al*, 2008). All the mutations altered residues that are phosphorylation sites for GSK-3*β* (codon 33), or are adjacent residues (codons 32, 34 and 40). These are predicted to prevent phosphorylation of CTNNB1 by GSK-3*β*, and therefore prevent its degradation.

In colon cancer, the WNT pathway is commonly activated by mutations in the mutation cluster region of *APC*. However, no mutations were found in this region in the CNS PNET or medulloblastoma cohorts. Further investigation to determine the molecular basis of pathway activation is needed to help understand if it is playing a role in disease development. It is possible that mutations are present in other regions of the *CTNNB1* or *APC* genes not investigated in this study. Alternative factors that could cause pathway activation in CNS PNETs include inactivating mutations in the pathway inhibitors *AXIN1* and *AXIN2*. Mutations have been identified in both genes in different tumour types including medulloblastoma, hepatocellular and colon carcinoma (Liu *et al*, 2000; Satoh *et al*, 2000; Taniguchi *et al*, 2002; Baeza *et al*, 2003; Koch *et al*, 2007). WNT ligands or their receptors could also be overexpressed. Earlier studies have identified increased expression of WNT and frizzled receptor genes in different tumour types (Huguet *et al*, 1994; Janssens *et al*, 2004; Merle *et al*, 2004). Epigenetic alterations have also been identified, including inactivation of secreted frizzled-related protein genes in colorectal cancer (Suzuki *et al*, 2004). Interaction of WNT/*β*-catenin signalling with other signalling pathways has been shown to affect the levels of signalling as well (Moon *et al*, 2002; Saldanha *et al*, 2004).

Although not statistically significant, survival analysis in the CNS PNET cohort suggested a trend towards a better prognosis for patients whose tumours displayed high CTNNB1 nuclear staining. Significance was achieved when high CTNNB1 nuclear tumours were compared with low CTNNB1 nuclear tumours, but was limited by the very small number of samples included in the analysis. Together with the differences in 5-year overall survival rates, the data suggested that a higher level of pathway activation was linked to a better outcome and is in agreement with the association of better prognosis with WNT/*β*-catenin pathway activation earlier found in medulloblastoma (Ellison *et al*, 2005; Gajjar *et al*, 2006). The number of samples included in this analysis was relatively low; therefore, further investigation is needed for confirmation of results.

No significant association was found for medulloblastomas in this study between pathway activation and survival. However, the Kaplan–Meier curves and overall survival rates suggest a trend towards better survival for patients with the pathway active in their tumours. Other clinical factors also suggest this, including the higher percentage of relapses in non-nuclear cases. Significance would need to be examined in a larger cohort.

Association of pathway activation with a favourable prognosis is somewhat surprising, considering its link to a poorer outcome and disease progression in other tumour types, including colon, hepatocellular and breast carcinoma (Lin *et al*, 2000; Inagawa *et al*, 2002; Bondi *et al*, 2004). However, an association with a better prognosis has been seen before in medulloblastoma and other tumour types such as non-small cell lung carcinoma and ovarian cancer (Hommura *et al*, 2002; Catasus *et al*, 2004; Ellison *et al*, 2005; Gajjar *et al*, 2006). There could be a number of reasons for this link in CNS PNET and medulloblastoma. Pathway activation could represent a subset of tumours with a less aggressive phenotype than other subtypes. Activation of the WNT/*β*-catenin pathway can have many different effects on a cell, including influencing proliferation, apoptosis and differentiation. Therefore, pathway activation could be causing a deleterious effect such as promoting apoptosis. Alternatively, pathway activation could affect sensitivity to treatment. A recent study in medulloblastoma cell lines demonstrated activation of the pathway after irradiation (Salaroli *et al*, 2008). The high proportion of tumours displaying pathway activation in both CNS PNET and medulloblastoma suggest that it could be an important treatment target. However, the reason for the association with favourable prognosis needs to be understood before strategies for targeting the pathway are developed.

A high proportion of the medulloblastomas displaying nuclear CTNNB1 staining were of the desmoplastic subtype, which differs from earlier results (Thompson *et al*, 2006). Desmoplastic tumours have been associated with a better survival than the classic subtype, which might suggest this is the cause of better prognosis in the nuclear positive cases in this study (Sure *et al*, 1995). No statistically significant association between desmoplastic cases and survival was found in this study. However, median survival for desmoplastic cases was greater than the rest of the cohort (12 *vs* 2 years), suggesting that the lack of statistical significance may be because of the small sample size (*n*=37). Median survival for desmoplastic cases with nuclear CTNNB1 staining was greater than desmoplastic cases with only cytoplasmic or negative staining (7 *vs* 3.5 years). Although not significant, this data suggests that tumour subtype was not the cause of better prognosis for the CTNNB1 nuclear tumours. The most comprehensive analysis of WNT status and survival in medulloblastomas did not include any desmoplastic tumours (Ellison *et al*, 2005). The results from this study suggest that future cohorts investigated should include this tumour subtype. It is interesting to note that all the medulloblastomas that contained mutations were of the classical subtype. However, only 10% of the samples sequenced were desmoplastic.

Association between WNT/*β*-catenin pathway activation and chromosome 6 loss has earlier been found in medulloblastoma (Clifford *et al*, 2006; Thompson *et al*, 2006). Copy number data generated from Affymetrix SNP chip (Affymetrix, Santa Clara, CA, USA) analysis for 12 of the CNS PNETs used in this study (six nuclear and six cytoplasmic CTNNB1) suggest that this is not the case for this tumour type. Only one tumour displaying cytoplasmic CTNNB1 staining had a loss of one copy of chromosome 6 (S Miller, unpublished data).

In summary, the WNT/*β*-catenin pathway has been found to be active in over one-third of CNS PNETs, suggesting that it plays an important role in the pathogenesis of this tumour type. The percentage of samples displaying pathway activation is similar to results seen in medulloblastoma. However, the method of activation seems to differ from mutation of exon 3 of *CTNNB1*. The data has also revealed a potential link between survival and the extent of pathway activation in CNS PNETs. Further investigation involving a larger cohort is now needed to determine this. The mechanism of activation, as well as the role the pathway is playing in the pathogenesis of these tumours also needs to be determined to better understand their biology, as well as help to decide how the pathway could be targeted as a part of future treatment strategies.

## Figures and Tables

**Table 1:** Clinical characteristics of CNS PNET (*n*=55, including 12 pineoblastomas) and medulloblastoma (*n*=62) patient cohorts

	**CNS PNET**	**Medulloblastoma**
Sex (male:female ratio)	1.1 : 1	2.6 : 1
Mean age at diagnosis (years)	5.9 (0.4–15.5 years)	7.2 (0–14.4 years)
Percentage of patients who have relapsed	45%	35%
Average time to relapse (years)	1.1	2.2
		
*Metastatic status* a		
M0	55%	60%
M1	4%	3%
M2	11%	5%
M3	15%	21%
M4	4%	3%
Unknown	13%	8%
		
Median survival (years)	1.8	5.3
Median progression-free survival (years)	0.8	4.1
Median follow-up for patients still alive (years)	3.9 (*n*=12)	6.5 (*n*=28)
		
*Resection*		
Partial	53%	48%
Complete	27%	40%
Unknown	20%	11%
		
*Treatment* b		
None	18%	3%
Chemotherapy	24%	19%
Radiotherapy	7%	10%
Chemotherapy and radiotherapy	40%	68%
Unknown	11%	0%
		
*Medulloblastoma subtype* c		
Classic	—	56%
Desmoplastic	—	24%
Anaplastic	—	11%
Large cell	—	5%
Medullomyoblastoma	—	3%

Abbreviation: CNS PNET=central nervous system primitive neuroectodermal tumour.

a Metastatic stages according to Chang staging system (Chang *et al*, 1969).

b Chemotherapy and radiotherapy was not uniform across all patients.

c According to the WHO criteria (Louis *et al*, 2007).

**Table 2:** Results for CNS PNET and medulloblastoma immunohistochemistry cohorts

**Case^a^**	**Location**	**Sex**	**Histological subtype^b^**	**CTNNB1 IHC**	**CCND1 IHC**	**CTNNB1 mutation^c^**
SP01	Cerebral	F	−	C	+	WT
SP02	Cerebral	F	−	C	−	WT
SP03	Cerebral	F	−	C	−	WT
SP04	Cerebral	M	−	C	−	WT
SP05	Cerebral	M	−	C	−	WT
SP06	Cerebral	F	−	C	−	WT
SP07	Pineal	M	−	C	−	WT
SP08	Pineal	F	−	C	−	WT
SP09	Cerebral	F	−	C	−	No result
SP10	Pineal	M	−	C	−	No result
SP11	Cerebral	F	−	C	−	No result
SP12	Cerebral	F	−	C	No result	No result
SP13	Cerebral	F	−	C	+	No result
SP14	Cerebral	F	−	O	+	No result
SP15	Cerebral	F	−	C	+	No result
SP16	Pineal	M	−	C	−	No result
SP17	Cerebral	M	−	C	−	No result
SP18	Cerebral	M	−	C	−	No result
SP19	Cerebral	M	−	N low	+	WT
SP20	Pineal	M	−	N low	−	No result
SP21	Cerebral	F	−	N low	+	No result
SP22	Cerebral	M	−	N low	−	No result
SP23	Cerebral	M	−	N high	+	No result
SP24	Cerebral	M	−	N high	+	WT
SP25	Cerebral	M	−	N high	+	WT
SP26	Cerebral	F	−	N high	+	WT
SP27	Cerebral	M	−	N high	+	No result
SP28	Cerebral	M	−	N high	+	No result
SP01R	Cerebral	F	−	C	+	No result
SP29R	Cerebral	M	−	C	+	WT
SP30R	Cerebral	F	−	C	+	No result
SP24R	Cerebral	M	−	N high	+	WT
SP31R	Cerebral	F	−	N high	+	No result
SP32R	Cerebral	M	−	N high	+	No result
MB01	Posterior fossa	M	Classical	C	−	WT
MB02	Posterior fossa	M	Anaplastic	C	−	WT
MB03	Posterior fossa	F	Desmoplastic	C	−	WT
MB04	Posterior fossa	M	Classical	C	−	WT
MB05	Posterior fossa	F	Classical	C	−	No result
MB06	Posterior fossa	F	Classical	C	−	No result
MB07	Posterior fossa	M	Anaplastic	O	−	No result
MB08	Posterior fossa	M	Classical	C	−	No result
MB09	Posterior fossa	M	Anaplastic	O	−	No result
MB10	Posterior fossa	F	Classical	C	−	No result
MB11	Posterior fossa	M	Desmoplastic	C	−	No result
MB12	Posterior fossa	M	Anaplastic	C	−	No result
MB13	Posterior fossa	F	Desmoplastic	O	−	No result
MB14	Posterior fossa	M	Large cell	C	−	No result
MB15	Posterior fossa	M	Anaplastic	C	−	No result
MB16	Posterior fossa	M	Desmoplastic	C	−	No result
MB17	Posterior fossa	M	Classical	C	−	No result
MB18	Posterior fossa	M	Classical	C	−	No result
MB19	Posterior fossa	M	Desmoplastic	C	−	No result
MB20	Posterior fossa	M	Classical	O	+	No result
MB21	Posterior fossa	F	Classical	O	−	No result
MB22	Posterior fossa	M	Large cell	C	−	No result
MB23	Posterior fossa	M	Classical	C	−	No result
MB24	Posterior fossa	M	Desmoplastic	C	−	No result
MB25	Posterior fossa	M	Desmoplastic	C	−	No result
MB26	Posterior fossa	F	Desmoplastic	C	−	No result
MB27	Posterior fossa	M	Anaplastic	C	−	No result
MB28	Posterior fossa	M	Desmoplastic	N low	+	No result
MB29	Posterior fossa	M	Desmoplastic	N low	−	No result
MB30	Posterior fossa	M	Classical	N low	−	No result
MB31	Posterior fossa	M	Medullomyoblastoma	N low	−	No result
MB32	Posterior fossa	M	Desmoplastic	N low	−	No result
MB33	Posterior fossa	M	Desmoplastic	N high	−	No result
MB34	Posterior fossa	F	Classical	N high	−	No result
MB35	Posterior fossa	M	Desmoplastic	N high	−	No result
MB36	Posterior fossa	M	Large cell	N high	+	No result
MB37	Posterior fossa	M	Desmoplastic	N high	+	No result
MB38R	Posterior fossa	M	Anaplastic	O	−	No result

Abbreviations: C=cytoplasmic staining; CNS PNET=central nervous system primitive neuroectodermal tumour; IHC=immunohistochemistry; N high=high nuclear staining; N low=low nuclear staining; MB=medulloblastoma; O=negative staining; WT=wild type; +=over expression; −=no expression.

a SP=CNS PNET. R indicates a recurrent sample. Primary and recurrent samples with the same case number indicate that samples are from the same patient.

b Histological subtype is only included for medulloblastoma samples, according to the WHO criteria (Louis *et al*, 2007).

c Additional samples were sequenced that were not included in the immunohistochemistry cohorts.
