# Supplementary material for: An investigation of WNT pathway activation and association with survival in central nervous system primitive neuroectodermal tumours (CNS PNET)
Source: Br J Cancer. 2009 Mar 17;100(8):1292–302. doi: 10.1038/sj.bjc.6604979 (PMC2676550; doi:10.1038/sj.bjc.6604979)
Supplement: Supplementary Tables 1–3 [file 6604979x1.doc]

**Supplemental Table 1.** Comparison of the clinical characteristics of scorable (n=28) and unscorable (n=14) primary tumour samples, for CTNNB1 IHC, from the CNS PNET cohort. Data from the whole CNS PNET cohort is displayed for comparison. Scorable and unscorable cohorts were comparable. The scorable cohort was also similar to the overall CNS cohort.

|  | **Scorable** | **Unscorable** | **Whole CNS PNET Cohort** |
| --- | --- | --- | --- |
| **Sex (male:female ratio)** | 1.2:1 | 1.3:1 | 1.1:1 |
| **Mean age at diagnosis (years)** | 5.8 (0.5 – 15.5 years) | 5.2 (0.4 – 14.9 years) | 5.9 (0.4 – 15.5 years) |
| **Percentage of patients who have relapsed** | 50% | 50% | 45% |
| **Average time to relapse (years)** | 1.1 | 1.3 | 1.1 |
| **Metastatic Status***  **M0**  **M1**  **M2**  **M3**  **M4**  **Unknown** | 68%  0%  11%  14%  7%  0% | 64%  0%  14%  21%  0%  0% | 55%  4%  11%  15%  4%  13% |
| **Median survival (years)** | 1.8 | 1.3 | 1.8 |
| **Median Progression Free Survival (years)** | 0.9 | 0.7 | 0.8 |
| **Resection**  **Partial**  **Complete**  **Unknown** | 71%  21%  7% | 50%  36%  14% | 53%  27%  20% |
| **Treatment^†^**  **None**  **Chemotherapy**  **Radiotherapy**  **Chemotherapy and radiotherapy**  **Unknown** | 14%  25%  7%  50%  4% | 29%  29%  14%  21%  7% | 18%  24%  7%  40%  11% |

*Metastatic stages according to Chang staging system (Chang et al., 1969)

^†^Chemotherapy and radiotherapy was not uniform across all patients

**Supplemental Table 2.** Comparison of the clinical characteristics of scorable (n=37) and unscorable (n=6) primary tumour samples, for CTNNB1 IHC, from the medulloblastoma cohort. Data from the whole medulloblastoma cohort is displayed for comparison. Scorable and unscorable cohorts were mostly comparable. The scorable cohort was also similar to the overall medulloblastoma cohort.

|  | **Scorable** | **Unscorable** | **Whole Medulloblastoma Cohort** |
| --- | --- | --- | --- |
| **Sex (male:female ratio)** | 3.6:1 | 2:1 | 2.6:1 |
| **Mean age at diagnosis (years)** | 6.9 (0.8 – 14.4 years) | 7.1 (0.9 – 12.8 years) | 7.2 (0 - 14.4 years) |
| **Percentage of patients who have relapsed** | 38% | 33% | 35% |
| **Average time to relapse (years)** | 2.4 | 3.5 | 2.2 |
| **Metastatic Status***  **M0**  **M1**  **M2**  **M3**  **M4**  **Unknown** | 62%  5%  8%  11%  5%  8% | 67%  0%  0%  33`%  0%  0% | 60%  3%  5%  21%  3%  8% |
| **Median survival (years)** | 3.5 | 0.5 | 5.3 |
| **Median Progression Free Survival (years)** | 2.1 | 0.3 | 4.1 |
| **Resection**  **Partial**  **Complete**  **Unknown** | 41%  46%  14% | 33%  33%  33% | 48%  40%  11% |
| **Treatment^†^**  **None**  **Chemotherapy**  **Radiotherapy**  **Chemotherapy and radiotherapy** | 3%  24%  14%  59% | 17%  0%  0%  83% | 3%  19%  10%  68% |
| **Subtype**^‡^  **Classic**  **Desmoplastic**  **Anaplastic**  **Large Cell**  **Medullomyoblastoma** | 35%  38%  16%  8%  3% | 100%  0%  0%  0%  0% | 56%  24%  11%  5%  3% |

*Metastatic stages according to Chang staging system (Chang et al., 1969)

^†^Chemotherapy and radiotherapy was not uniform across all patients

^‡^According to the WHO criteria (Louis et al., 2007)

**Supplemental Table 3.** Comparison of the clinical characteristics of scorable (n=18) and unscorable (n=24) primary tumour samples, for MKI67 IHC, from the CNS PNET cohort. Data from the whole CNS PNET cohort is displayed for comparison. Scorable and unscorable cohorts were comparable. The scorable cohort was also similar to the overall CNS PNET cohort.

|  | **Scorable** | **Unscorable** | **Whole CNS PNET Cohort** |
| --- | --- | --- | --- |
| **Sex (male:female ratio)** | 1.6:1 | 1:1 | 1.1:1 |
| **Mean age at diagnosis (years)** | 6.4 (1.0 – 15.5 years) | 5 (0.4 – 15.3 years) | 5.9 (0.4 – 15.5 years) |
| **Percentage of patients who have relapsed** | 44% | 54% | 45% |
| **Average time to relapse (years)** | 1.5 | 0.9 | 1.1 |
| **Metastatic Status***  **M0**  **M1**  **M2**  **M3**  **M4**  **Unknown** | 72%  0%  11%  6%  11%  0% | 63%  0%  13%  25%  0%  0% | 55%  4%  11%  15%  4%  13% |
| **Median survival (years)** | 1.8 | 1.3 | 1.8 |
| **Median Progression Free Survival (years)** | 1.5 | 0.7 | 0.8 |
| **Resection**  **Partial**  **Complete**  **Unknown** | 67%  33%  0% | 63%  21%  17% | 53%  27%  20% |
| **Treatment^†^**  **None**  **Chemotherapy**  **Radiotherapy**  **Chemotherapy and radiotherapy**  **Unknown** | 17%  22%  6%  50%  6% | 21%  29%  13%  33%  4% | 18%  24%  7%  40%  11% |

*Metastatic stages according to Chang staging system (Chang et al. 1969)

^†^Chemotherapy and radiotherapy was not uniform across all patients
